# Emotional Intelligence in Patients with Spinal Cord Injury (SCI)

**Published:** 2017-05

**Authors:** Hooshang SABERI, Mahsa GHAJARZADEH

**Affiliations:** 1. Dept. of Neurosurgery, Brain and Spinal Cord Injury Research Center, Tehran University of Medical Sciences, Tehran, Iran; 2. Brain and Spinal Injury Research Center, Tehran University of Medical Sciences, Tehran, Iran; 3. Universal Council of Epidemiology (UCE), Universal Scientific Education and Research Network (USERN), Tehran, Iran

**Keywords:** SCI, Emotional intelligence, Injury level

## Abstract

**Background:**

Spinal Cord Injury (SCI) is a devastating situation. Spinal Cord Injury affects functional, psychological and socioeconomic aspects of patients’ lives. The ability to accomplish and explicate the one’s own and other’s feelings and emotions to spread over appropriate information for confirming thoughts and actions is defined as emotional intelligence (EI). The goal of this study was to evaluate depression and EI in SCI patients in comparison with healthy subjects.

**Methods:**

One-hundred-ten patients with SCI and 80 healthy subjects between Aug 2014 and Aug 2015 were enrolled. The study was conducted in Imam Hospital, Tehran, Iran. All participants were asked to fill valid and reliable Persian version Emotional Quotient inventory (EQ-i) and Beck Depression Inventory (BDI). All data were analyzed using SPSS. Data were presented as Mean±SD for continuous or frequencies for categorical variables. Continuous variables compared by means of independent sample *t*-test. *P*-values less than 0.05 were considered as significant.

**Results:**

Mean age of patients was 28.7 and mean age of controls was 30.2 yr. Spinal cord injury in 20 (18.3%) were at cervical level, in 83 (75.4%) were thoracic and in 7 (6.3%) were lumbar. Mean values of independence, stress tolerance, self-actualization, emotional Self-Awareness, reality testing, Impulse Control, flexibility, responsibility, and assertiveness were significantly different between cases and controls. Mean values of stress tolerance, optimism, self-regard, and responsibility were significantly different between three groups with different injury level. Most scales were not significantly different between male and female cases.

**Conclusion:**

Emotional intelligence should be considered in SCI cases as their physical and psychological health is affected by their illness.

## Introduction

Spinal cord injury (SCI) is a devastating situation that affects 2.1–57.8 cases per million each year (1). It is associated with more or less disability depending on the level of injury (2). It affects functional, psychological and socioeconomic aspects of patients’ lives. Therefore, patients with SCI experience major impairments in all aspects of their lives. Accidents, sports, violence, falling and heavy dropping are major causes of traumatic SCI. Level and degree of the injury will cause various range of morbidities.

Emotional problems such as depression, anxiety, and post-traumatic stress disorder (PTSD) are common in these patients (3).

The ability to accomplish and explicate the one’s own and other’s feelings and emotions to spread over appropriate information for confirming thoughts and actions is defined as emotional intelligence (EI)(4). Perception, understanding, and regulation of emotions are essential components for controlling emotions and EI (5, 6). Although psychological dimensions followed by SCI should be considered in patients with SCI, there is no study considering EI in these patients.

These patients after injury do not participate in social programs and they prefer to be at home. Therefore, their social well-being will be affected which could affect personal and interpersonal relationships. We have designed this study to evaluate depression and EI in SCI patients in comparison with healthy subjects.

## Material and Methods

This cross-sectional study has been conducted in Spinal Cord Injury Clinic of Imam Hospital, Tehran University of Medical Sciences, Tehran, Iran between Aug 2014 and Aug 2015. One hundred and ten patients with SCI and 80 healthy subjects (the patient’s relatives were enrolled as controls). The participants filled the informed consent forms. The study was approved by local Ethics Committee.

Demographic data (sex, age), duration of the injury, marital status and level of injury were recorded for all patients. All participants were asked to fill valid and reliable Persian version of Emotional Quotient inventory (EQ-i) and Beck Depression Inventory (BDI).

Emotional Quotient inventory (EQ-i) included 90 questions (7, 8). It is a self-report questionnaire, which includes 5 categories and 15 scales. The five categories are Intrapersonal (Self-Regard, Emotional Self-Awareness, Assertiveness, Independence, and Self Actualization), Interpersonal Empathy, Social Responsibility, and Interpersonal Relationship), Stress Management (Stress Tolerance and Impulse Control), Adaptability (Reality Testing, Flexibility and Problem Solving), and General Mood Scale (Optimism and Happiness). Each question is based on a 5-point Likert scale scoring system ranging from 5 to 1 (completely agree: 5 to completely disagree: 1). The total score is the sum of all questions scores. Higher score is indicative of higher emotional intelligence (6).

Beck Depression Inventory (BDI) should have been answered according to the patient’s feelings in the last week including 21 questions. Each answer scores from 0–3 to determine how depressed a person is. Individuals with scores between 0 and 9 are not recognized as depressed, scores between 10 and 18 indicate mild to moderate depression, scores between 19 and 29 values indicate individuals with moderate to severe depression, and scores between 30 and 63 correspond to severe depression(9).

### Statistical analysis

All data were analyzed using SPSS software version 20 (SPSS Inc., Chicago, IL, USA). Data were presented as Mean± SD for continuous or frequencies for categorical variables. Continuous variables compared by means of independent sample t-test. *P*-value less than 0.05 were considered as significant.

## Results

Mean age of patients was 28.7 and mean age of controls was 30.2 (*P*=0.3). Ninety-six (87.3%) patients were male and 14 (12.7%) were female while 69 (86.2%) controls were male and 11 (13.7%) were female. Spinal cord injury in 20 (18.3%) were at cervical level, in 83 (75.4%) were thoracic and in 7 (6.3%) were lumbar. Mean values of independence, stress tolerance, self-actualization, emotional self-awareness, reality testing, impulse control, flexibility, responsibility, and assertiveness were significantly different between cases and controls (Table 1).

**Table 1:** Mean score of EI subscale between patients and controls

**Subscale**	**Controls**	**Patients**	***P*-value**
Problem solving	13.2±2.8	12.9±3.1	0.5
Happiness	14.2±4.7	12.6±4.5	0.02
Independence	16.6±3.1	13.1±4.6	<0.001
Stress Tolerance	17±4	15±4.8	0.003
Self-Actualization	14.4±3.6	12.2±4.1	<0.001
Emotional Self-Awareness	15.3±2.8	13±3.8	<0.001
Reality Testing	17.4±3.3	14.6±4	<0.001
Interpersonal Relationship	12.1±3.2	11.9±3.5	0.7
Optimism	12.7±3.8	12.5±3.9	0.6
Self-Regard	13.3±4.1	12.2±4.1	0.08
Impulse Control	18.3±4.5	16.4±5.2	0.009
Flexibility	17.4±3.7	16.2±3.9	0.04
Responsibility	12.4±3.2	11.3±2.6	0.04
Empathy	11.6±2.7	12±3.1	0.3
Assertiveness	15.4±2.7	14.2±3.9	0.02

Mean values of stress tolerance, optimism, self-regard, and responsibility were significantly different between three groups with different injury level (Table 2). Most scales were not significantly different between male and female cases (Table 3).

**Table 2:** Comparison of scores between cases with different injury levels

**Subscale**	**Lumbar**	**Thoracic**	**Cervical**	***P*-value**
Problem solving	14.2±2.5	12.9±3	13.5±3	0.1
Happiness	15.9±5.4	13.8±4.6	13.1±1	0.1
Independence	17.9±2	16.4±3.4	15.8±1	0.1
Stress Tolerance	19.3±2.3	16.3±4.3	18.5±0.5	0.007
self-Actualization	14.9±4.3	14.3±3.6	14.5±0.5	0.8
Emotional Self-Awareness	14.9±3.1	15.4±2.9	15.4±0.4	0.7
Reality Testing	16.9±2.8	17.7±3.5	15.5±0.5	0.1
Interpersonal Relationship	12.4±3.5	11.9±3.2	13.5±0.5	0.3
Optimism	15.6±4.9	12.1±3.3	12.5±0.5	0.001
Self-Regard	16.3±4	12.4±3.9	14.8±2.6	<0.001
Impulse Control	18.8±4.8	18.5±4.6	15	0.1
Flexibility	18.9±3.4	17±3.9	18.2±3.6	0.09
Responsibility	13.9±3.8	10±3	10.5±0.5	0.01
Empathy	12±1.9	11.4±3	12	0.6
Assertiveness	15.1±3	15.5±2.7	15	0.7

**Table 3:** Comparison of scales between male and female ones

**Subscale**	**Men**	**Women**	***P* value**
Problem solving	13.1±2.9	13.6±2.7	0.5
Happiness	13.9±4.7	15.8±4.2	0.1
Independence	16.4±3.2	18.1±2.2	0.06
Stress Tolerance	16.6±3.9	20±3.9	0.003
Self-Actualization	14.1±3.7	16.8±2.2	0.001
Emotional Self-awareness	15.1±2.9	16.7±1.5	0.005
Reality Testing	17.1±3.2	19.6±3.2	0.009
Interpersonal Relationship	12.1±3.2	11.8±3	0.7
Optimism	12.5±3.8	14.1±3.9	0.1
Self-Regard	13.2±4.1	13.7±4.2	0.6
Impulse Control	18.1±4.6	19.7±3.7	0.2
Flexibility	17.2±3.9	18.7±2.3	0.05
Responsibility	10.4±3.3	10.4±2.3	0.9
Empathy	11.7±2.7	10.9±3	0.3
Assertiveness	15.1±2.6	17±2.9	0.01

## Discussion

The results showed that mean values of independence, stress tolerance, self-actualization, emotional self-awareness, reality testing, Impulse control, flexibility, responsibility, and assertiveness were significantly different between cases and controls. Emotional intelligence of patients with SCI is affected and perception, understanding, and regulation of emotions in patients with SCI are impaired. We also found that mean values of different subscale of EI except for interpersonal relationship, responsibility and empathy were significantly different between SCI cases and controls.

In this study, mean values of stress tolerance, optimism, self-regard, and responsibility were significantly different between three groups and the scores in cases with lumbar injuries were significantly higher. This could be indicative of effects of injury level on person’s life.

In this study, mean values of stress tolerance, self-actualization, emotional self-awareness, reality testing and assertiveness subscales were significantly different between male and female participants as well as significant difference between responsibility and empathy subscales of male and female MS cases (6). Women deal with spinal cord injury better than men. We investigated similar scores of EI in female and male medical residents of Tehran university of medical sciences (except responsibility) (4). Emotional intelligence of nursing students was evaluated and reported no significant difference between male and female ones (10). In healthy ones, there is no significant difference between EI score while patients with different diseases show different results.

Spinal cord injury is a devasting health condition which affects all aspects of patient’s lives. Therefore physical, emotional, financial and social parameters of SCI cases are affected (11, 12). Due to SCI complications such as loss of continence, SCI patients become dependent on their relatives and according to the extent of injury, and current life situation they will have wide range of psychological and mental problems. High prevalence of depression, psychological distress, and psychological morbidity may be observed in SCI cases (13–14). Therefore, their psychological health is affected. Emotional intelligence includes perception, understanding, and regulation of emotions derived from the concept that intelligence is multidimensional (5) and includes components such as social, emotional and behavioral aspects. Higher EI is associated with better performance.

## Conclusion

Emotional intelligence should be considered in SCI cases as their physical and psychological health are affected by their illness.

## Ethical considerations

Ethical issues (Including plagiarism, informed consent, misconduct, data fabrication and/or falsification, double publication and/or submission, redundancy, etc.) have been completely considered by the authors.
